# Neonatal screening and genotype-phenotype correlation of hyperphenylalaninemia in the Chinese population

**DOI:** 10.1186/s13023-021-01846-w

**Published:** 2021-05-12

**Authors:** Xin Wang, Yanyun Wang, Dingyuan Ma, Zhilei Zhang, Yahong Li, Peiying Yang, Yun Sun, Tao Jiang

**Affiliations:** Genetic Medicine Center, Nanjing Maternity and Child Health Care Hospital, Women’s Hospital of Nanjing Medical University, 123 Tianfei St., Qinhuai District, Nanjing, 210004 People’s Republic of China

**Keywords:** Hyperphenylalaninemia, Neonatal screening, Phenylalanine hydroxylase deficiency, Tetrahydrobiopterin deficiency

## Abstract

**Background:**

Hyperphenylalaninemia (HPA) is the most common amino acid metabolic disease involving phenylalanine hydroxylase (PAH, OMIM*612,349) deficiency or coenzyme tetrahydrobiopterin (BH4) deficiency. Patients with severe HPA often have a difficult life. Early diagnosis of HPA before the development of symptoms is possible via neonatal screening, facilitating appropriate treatment and reducing mortality and disability rates. This study revealed the prevalence, mutational and phenotypic spectrum, and prognosis of HPA by neonatal screening from January 2001 to September 2020 in Nanjing, Jiangsu Province, China.

**Methods:**

Through a retrospective analysis of the information available in the neonatal screening database, the clinical presentations, laboratory data, molecular characteristics and treatment follow-up data of HPA patients detected by neonatal screening were evaluated.

**Results:**

We diagnosed 181 patients with HPA from 1 to 957 newborns, giving an incidence of 1:6873. Among these patients, 177 were identified as PAH deficient and four patients were BH4 deficient. The average current age of the patients was 6.38 years old. The most common mutations of *PAH* were c.728 C > A/ p.Arg243Gln (13.83 %), c.158G > A/ p.Arg53His (9.57 %), c.611 A > G/ p.Tyr204Cys (7.44 %), and c.721 C > T/ p.Arg241Cys (6.38 %).

**Conclusions:**

This study revealed the prevalence, phenotype-genotype, and prognosis of HPA in China and contributes to the updating of PAHD data for China and worldwide. Our study not only expanded the spectrum of phenotypes and genotype but also provided a valuable tool for improved genetic counseling and management of future cases.

**Supplementary Information:**

The online version contains supplementary material available at 10.1186/s13023-021-01846-w.

## Background

Hyperphenylalaninemia (HPA), which is the most commonly occurring genetic amino acid metabolism disorder, is caused by enzyme defects in the phenylalanine metabolic pathway, such as phenylalanine hydroxylase (PAH) deficiency (PAHD) (OMIM 261,600) and coenzyme tetrahydrobiopterin deficiency (BH4D), which lead to elevated serum phenylalanine (Phe) concentrations [1]. Serious clinical manifestations of HPA include irreversible brain damage, intellectual deficiency, and epilepsy [2]. However, the early initiation of therapy is remarkably successful in preventing these severe neurological features and ensuring healthy growth [3].

Human *PAH*, with a total length of 1.5 Mb, is located on chromosome 12q22-q24.1. There are over 1,000 pathogenic *PAH* mutations (http://www.biopku.org/pah), which show obvious racial and regional differences, for example, p.Arg408Trp is the most common mutation in Eastern Europe, and IVS10-11G > A is the most common in Middle East regions such as Iran [4].

According to the severity of HPA, PAHD phenotype classifications range from mild hyperphenylalaninemia (MHP, serum Phe concentration: 120–360 µmol/L, only regular follow-up is needed) through mild phenylketonuria (mPKU, serum Phe concentration: 360–1200 µmol/L) to classic phenylketonuria (cPKU, serum Phe concentration: ≥1200 µmol/L). This broad phenotype range arises from different degrees of PAH enzyme activity reduction, and both mPKU and cPKU patients require dietary therapy [5, 6].

BH4D accounts for approximately 1–2 % of HPA cases [7, 8]. Five enzymes are involved: GTP cyclohydrolase I, 6-pyruvoyl-tetrahydropterin synthase (PTPS), sepiapterin reductase, pterin-4-carbinolamine dehydratase, and dihydropteridine reductase (DHPR). BH4D affects the synthesis of neurotransmitters such as dopamine and serotonin [7], and the resulting deficiency leads not only to elevated serum Phe concentrations but also to neurological symptoms and signs, and sometimes death [9–11]. BH4D was once known as “malignant PKU” in China [12].

In recent years, a gene associated with HPA, *DNAJC12*, was identified [13]. The protein encoded by *DNAJC12* acts together with 70-kDa heat shock protein (HSP70) to induce the proper folding of phenylalanine hydroxylase. The clinical symptoms triggered by mutations of *DNAJC12* include mild autistic features, hyperactivity to severe intellectual deficiency, dystonia, and parkinsonism [14]. The discovery of this gene has opened up new opportunities for the genetic diagnosis of HPA.

Neonatal screening for HPA in China has been performed since the early 1980 s, and considerable experience has accumulated after more than 30 years. In 2014, Chinese experts proposed “a consensus for the diagnosis and treatment of HPA” [15]. Furthermore, in 2019, “a consensus for diet treatment and nutrition management of PAHD” was also recommended [16]. Our hospital was one of the first in China to be involved in this scheme, and we have been carrying out neonatal HPA screening for 35 years. In this study, a review of the last 20 years of data was performed to investigate the latest developments in patients currently associated with HPA. The primary objectives of the study were to analyze correlations between the genotypes and phenotypes of PAHD patients, determine the blood Phe control characteristics of PAHD patients over the past 4 years, and assess the prognoses based on current treatment guidelines and therapeutic goals.

## Results

### Demographics and disease characterization

In total, 1 292 622 newborns from Nanjing, Jiangsu Province, China, were screened for HPA from January 2001 to September 2020. We diagnosed 181 cases of HPA, and the incidence rate was 1 in 6 873 (Table 1). The positive primary neonatal screening rate was 0.087 %, and when babies were recalled, about 100 % revisited. The positive predictive value (PPV) was approximately 9.09 % (Additional file 1: Table S1). All patients were treated before they reached 1 month old, and the mean age for initiation treatment was 20 ± 7 days after birth.

**Table 1:** Statistical summary of HPA incidence, as detected by neonatal screening

Time frame	Neonate	Screening	Positive	Incidence
2001–2005	211 510	184 103 (86.14 %)	12	1:15,342 (0.0065 %)
2006–2010	282 041	270 477(95.83 %)	36	1:7513 (0.0133 %)
2011–2015	383 437	377 434 (98.38 %)	58	1:6507 (0.0154 %)
2016–2020	415 634	411 943 (99.12 %)	75	1:5493 (0.0182 %)
2001–2020	1 292 622	1 243 957 (96.24 %)	181	1:6873 (0.0146 %)

Positive, patients diagnosed with HPA

## Clinical classification

Among the 181 HPA patients, 100 patients were male and 81 patients were female, with a male-to-female ratio of 1.2:1. As shown in Additional file 1: Tables S2, 177 patients (97.79 %) were identified as having PAHD and four patients (2.21 %) were identified as having BH4D. All four BH4D patients were PTPS deficient. The urinary pterin profile analysis results are provided in Additional file 1: Table S3.

**Fig. 1:**
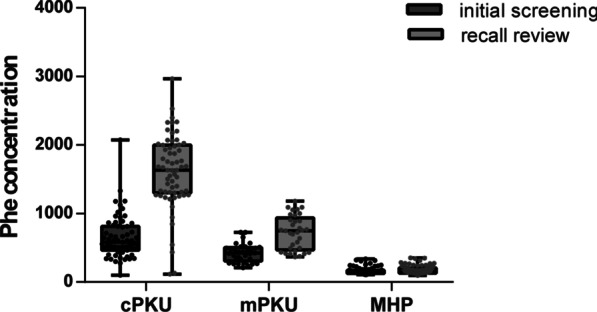
Serum Phe concentrations for cPKU (n = 63), mPKU (n = 33), and MHP (n = 81) patients in initial screening and recall review. ***, P < 0.001; NS, no significant difference; error bars represent SEM

Of the 177 PAHD patients, 63 patients had cPKU, with Phe concentrations of 651.17 ± 308.92 µmol/L (initial screening) and 1611.47 ± 532.61 µmol/L (recall review). Thirty-three patients had mPKU, having Phe concentrations of 423.94 ± 130.96 µmol/L (initial screening) and 725.44 ± 243.61 µmol/L (recall review). A further 81 patients had MHP and showed Phe concentrations of 160.09 ± 51.74 µmol/L (initial screening) and 170.84 ± 59.44 µmol/L (recall review) (Additional file 1: Table S2). The results showed that the cPKU patients had significantly higher Phe concentrations than the mPKU and MHP patients (P < 0.001), and the mPKU patients showed higher Phe concentrations than patients with MHP (P < 0.001). Furthermore, in the cPKU and mPKU patients, the Phe concentrations at the recall review were significantly higher than those at the initial screening (P < 0.001), whereas there was no significant difference in MHP patients’ Phe concentrations between the initial screening and recall review (Fig. 1).

## Gene variant and allele distributions

Of the 181 HPA patients, 48 agreed to genetic analysis (47 with PAHD and 1 with BH4D; 28 males and 20 females); they came from 48 unrelated Han families, and none of their parents were consanguineous. Variant analysis was performed after clinical diagnosis, about 1–3 months after birth.

There were 40 mutations detected in the 47 PAHD patients, included 37 mutations on *PAH*, one mutation on *PST* and two unspecified single nucleotide variations (SNVs), with the most common being c.728G > A (p.Arg243Gln; 13.83 %), c.158G > A (p.Arg53His; 9.57 %), c.611 A > G (p.Tyr204Cys; 7.44 %), and c.721 C > T (p.Arg241Cys; 6.38 %) (Table 2). In the BH4D (PTPS) patient, two mutations were detected in E5, including c.272 A > G (p.Lys91Arg) and c.286G > A (p.Asp96Asn).

**Table 2:** PAHD variants and allele distributions in PAHD patients

Index	Exon /intron	Nucleotide change	Amino acid change	Variant type	Allele frequency
1	E2	c.158G > A	p.Arg53His	Missense	9/94(9.57 %)
2	E3	c.194T > C	p.Ile65Thr	Missense	1/94(1.06 %)
3	E3	c.223G > C	p.Asp75His	Missense	1/94(1.06 %)
4	E3	c.301G > A	p.Asp101Asn	Missense	2/94(2.13 %)
5	E3	c.331 C > T	p.Arg111Ter	Nonsense	3/94(3.19 %)
6	E5	c.464G > A	p.Arg155His	Missense	1/94(1.06 %)
7	E5	c.472 C > T	p.Arg158Trp	Missense	1/94(1.06 %)
8	E5	c.494 C > A	p.Ala165Asp	Missense	1/94(1.06 %)
9	E5	c.505 C > A	p.Arg169Ser	Missense	1/94(1.06 %)
10	E6	c.527G > A	p.Arg176Gln	Missense	2/94(2.13 %)
11	E6	c.611 A > G	p.Tyr204Cys	Splice	7/94(7.44 %)
12	E6	c.649T > C	p.Cys217Arg	Missense	1/94(1.06 %)
13	E6	c.659 A > C	p.His220Pro	Missense	1/94(1.06 %)
14	E7	c.688G > A	p.Val230Ile	Missense	1/94(1.06 %)
15	E7	c.721 C > T	p.Arg241Cys	Missense	6/94(6.38 %)
16	E7	c.722G > C	p.Arg241Pro	Missense	1/94(1.06 %)
17	E7	c.722delG	p.Arg241fs	In-frame	1/94(1.06 %)
18	E7	c.728G > A	p.Arg243Gln	Missense	13/94(13.83 %)
19	E7	c.755G > A	p.Arg252Gln	Missense	1/94(1.06 %)
20	E9	c.964G > A	p.Ala322Thr	Missense	1/94(1.06 %)
21	E9	c.965 C > A	p.Ala322Asp	Missense	1/94(1.06 %)
22	E10	c.971T > A	p.Ile324Asn	Missense	1/94(1.06 %)
23	E10	c.1045T > G	p.Ser349Ala	Missense	4/94(4.26 %)
24	E11	c.1068 C > A	p.Tyr356Ter	Nonsense	2/94(2.13 %)
25	E11	c.1123 C > G	p.Glu375Glu	Missense	1/94(1.06 %)
26	E11	c.1174T > A	p.Phe392Ile	Missense	4/94(4.26 %)
27	E11	c.1197 A > T	p.Val399=	Missense	2/94(2.13 %)
28	E12	c.1222 C > T	p.Arg408Trp	Missense	1/94(1.06 %)
29	E12	c.1238G > C	p.Arg413Pro	Missense	5/94(5.32 %)
30	E12	c.1243G > A	p.Asp415Asn	Missense	1/94(1.06 %)
31	E12	c.1301 C > A	p.Ala434Asp	Missense	3/94(3.19 %)
32	I1	c.84-291 A > G		Splice	2/94(2.13 %)
33	I2	c.168 + 5G > C		Splice	1/94(1.06 %)
34	I3	c.353-2 A > T		Splice	1/94(1.06 %)
35	I4	c.442-1G > A		Splice	3/94(3.19 %)
36	I5	c.509 + 5delG		Splice	1/94(1.06 %)
37	I12	c.1315 + 5G > C		Splice	1/94(1.06 %)
38#	E2	c.155 A > G	p.Asn52Ser	Missense	1/94(1.06 %)
39*	E7	c.735G > A	p.Val245=	SNV	1/94(1.06 %)
40*	I4	c.61-907T > C		SNV	1/94(1.06 %)
41^0^	-	-		Undetected	2/94(2.13 %)
Total					94

# mutation on *PTS*; * Not specified variants site; ^0^ undetected variants

## Relationship between genotype and phenotype

We then focused on the relationships between the PAHD genotypes and biochemical phenotypes. Among the 40 mutations detected, c.728G > A (p.Arg243Gln) was homozygous in three cPKU patients and heterozygous in three cPKU patients, one mPKU patient, and three MHP patients; c.611 A > G (p.Tyr204Cys) was heterozygous in three cPKU patients, two mPKU patients, and two MHP patients; c.721 C > T (p.Arg241Cys) was heterozygous in one cPKU patient, two mPKU patients, and three MHP patients; whereas c.158G > A (p.Arg53His) was heterozygous and only found in eight MHP patients (Table 3). The serum Phe concentrations of patients with the mutation c.158G > A fluctuated between 94 and 145 µmol/L.

**Table 3:** Genotypes in each clinical phenotype

Clinical phenotype	Confirmatory level of Phe (µmol/L)	Genotype	Number of patients
cPKU	1849.1 ± 454.16	p.Arg243Gln / p.Arg243Gln	3/47
		p.Arg241Pro / p.Arg243Gln	1/47
		p.Arg241Cys / c.442-1G > A	1/47
		p.Arg413Pro/ -	1/47
		p.Arg413Pro/ p.Arg413Pro	1/47
		p.Tyr204Cys/ -	1/47
		p.Tyr204Cys/ p.Arg413Pro	1/47
		p.Arg111Ter / p.Ser349Ala	1/47
		p.Ile324Asn / p.Arg243Gln	1/47
		p.Arg252Gln/ c.1315 + 5G > C	1/47
		c.168 + 5G > C/ p.Tyr204Cys	1/47
		p.Ala434Asp/ c.353-2 A > T	1/47
		p.Arg111Ter / p.Arg243Gln	1/47
mPKU	650.0 ± 242.99	p.Tyr204Cys/ p.Ala434Asp	1/47
		p.Tyr204Cys/p.Ser349Ala	1/47
		p.Arg241Cys /p.Arg408Trp	1/47
		p.Arg243Gln / p.Arg241Cys	1/47
		p.Ile65Thr / p.Val399=	1/47
		p.Arg111Ter /p.Asp415Asn	1/47
		c.84-291 A > G / c.84-291 A > G	1/47
MHP	151.7 ± 36.88	p.Arg53His / p.Arg158Trp	1/47
		p.Arg53His / p.Arg241Cys	1/47
		p.Arg53His / p.Tyr204Cys	1/47
		p.Arg53His / p.Ser349Ala	1/47
		p.Arg53His / c.442-1G > A	1/47
		p.Arg53His / p.Arg243Gln	1/47
		p.Arg53His / p.Ala165Asp	1/47
		p.Arg53His / c.509 + 5delG	1/47
		p.Arg241Cys / p.Ser349Ala	1/47
		p.Arg241Cys / p.Ala322Thr	1/47
		p.Phe392Ile/ p.Ala322Asp	2/47
		p.Arg155His / p.Ala434Asp	1/47
		p.Val399=/ p.Asn52Ser	1/47
		c.442-1G > A/ p.Arg176Gln	1/47
		p.Tyr356Ter/ p.Phe392Ile	2/47
		p.Asp75His / p.His220Pro	1/47
		p.Arg243Gln / p.Val230Ile	1/47
		p.Arg169Ser / p.Arg243Gln	1/47
		p.Arg241fs / p.Asp101Asn	1/47
		p.Asp101Asn / p.Tyr204Cys	1/47
		p.Arg413Pro/ p.Arg176Gln	1/47
		c.61-907T > C/ p.Val245=	1/47
		p.Cys217Arg / p.Glu375Glu	1/47

-, undetected mutation

## Treatment and patient outcome

Patients with serum Phe of ≥ 360 µmol/L (n = 100) who required treatment with a low-Phe diet included those with cPKU, mPKU, and BH4D. During the study period, 12 patients ceased treatment because of economic or other factors, and these comprised nine cPKU patients (six theoretical aged ≥ 6 years old till now, three aged < 6 years old who refused treatment after diagnosed and died within 3 months after birth), one mPKU patient (aged ≥ 6 years old), and two with BH4D. Among these 12 cases, five died (four cPKU patients and one BH4D patient, Additional file 1: Table S4) and seven suffered intellectual deficiency (including one complicated with epilepsy). The other 88 patients received treatment; however, 21 of them were lost to follow-up during the course of treatment, including 15 cPKU patients (aged ≥ 6 years old) and six mPKU patients (two aged < 6 years old and four aged ≥ 6 years old). The remaining 67 patients (39 cPKU, 26 mPKU, and two BH4D) persisted with the follow-up treatment. Additionally, there were 29 MHP patients (24 aged < 6 years old and five aged ≥ 6 years old) who maintained regular revisits.

Among the two BH4D patients, one male stopped medication before 1 year of age, and his last follow-up age was 13, and one female received only a low-Phe diet, and her last follow-up age was 5. The growth and intellectual development of these two patients were within the normal ranges, and they showed normal brain function on EEG.

We reviewed the Phe concentrations and Phe/Tyr ratios in the follow-up data of the 94 patients (39 cPKU, 26 mPKU, and 29 MHP) recorded over the last 4 years (from 2017 to 2020), and their mean Phe concentration and Phe/Tyr ratio were 289.88 ± 15.64 µmol/L and 5.30 ± 0.83, respectively (Additional file 1: Table S5).

**Fig. 2:**
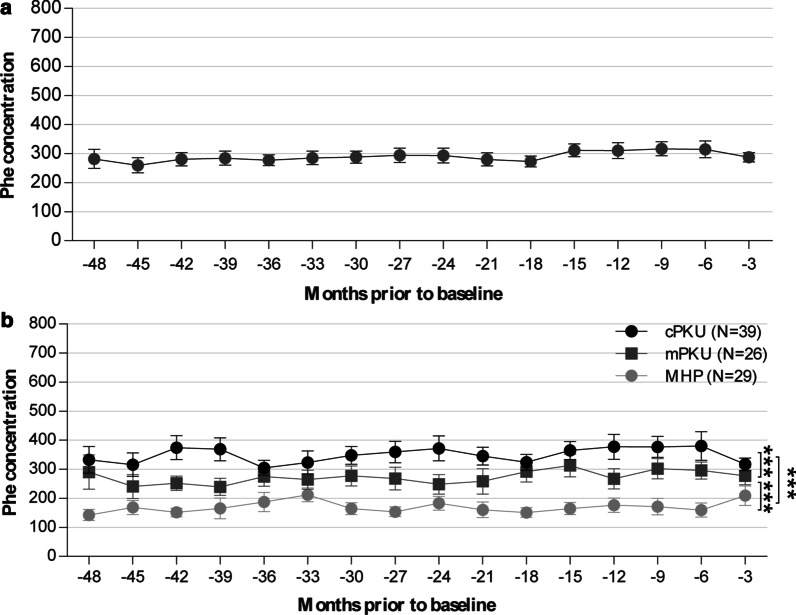
Mean serum Phe concentrations for PAHD patients over time across time windows of 3-month intervals. (A) Mean Phe (µmol/L) of PAHD patients (n = 94) over time. (B) Respective mean Phe (µmol/L) of cPKU, mPKU, and MHP patients over time. ***, P < 0.001; Error bars represent SEM

Although the Phe concentrations fluctuated around 300 µmol/L over the past 4 years, the Phe/Tyr ratio had a tendency to increase year by year, e.g., from 4 to 6 (Fig. 2 A and Additional file 1: Fig. S1A). The average Phe concentrations and Phe/Tyr ratios of cPKU patients were higher than those of the mPKU and MHP patients (P < 0.001), and those of mPKU patients were higher than those of MHP patients (P < 0.001) (Fig. 2B and Additional file 1: Fig. S1B).

**Fig. 3:**
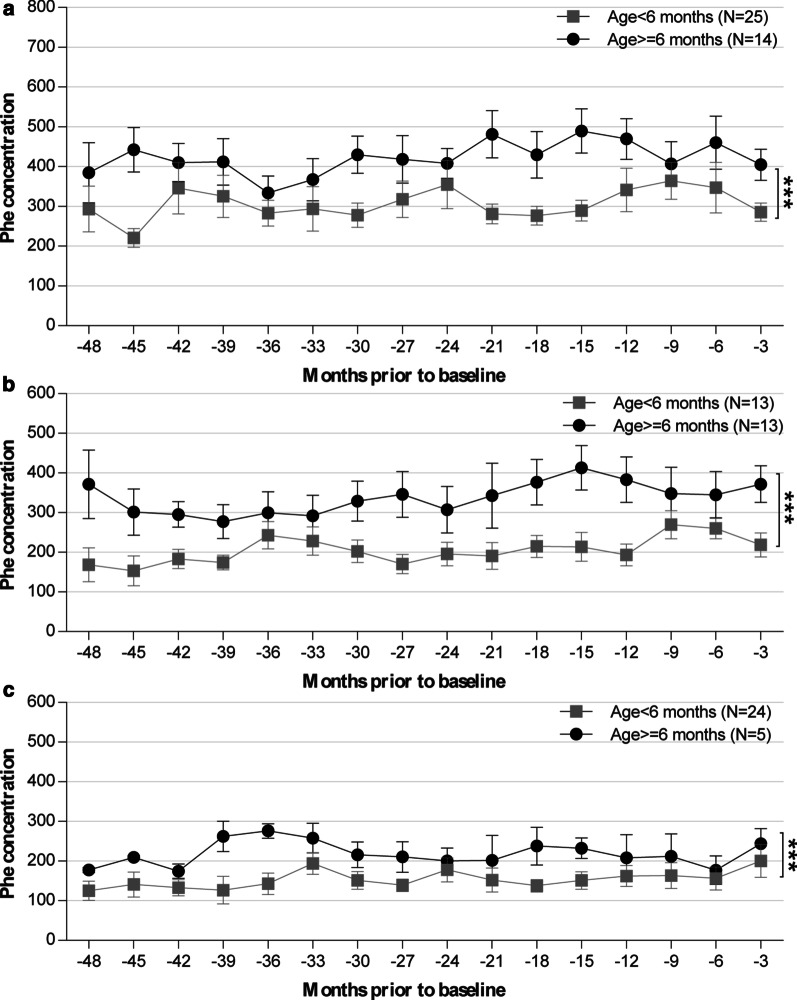
Mean serum Phe concentrations over time by age group (age < 6 years vs. age ≥ 6 years) for cPKU (A), mPKU (B), and MHP (C) patients. ***, P < 0.001; error bars represent SEM

We also divided these PAHD follow-up patients into preschool children (aged < 6 years old) and school-age children (aged ≥ 6 years old), and we found that school-age children tended to have higher Phe levels and Phe/Tyr ratios than preschool-age children (P < 0.001) (Fig. 3 and Additional file 1: Fig. S2).

The latest follow-up data showed that the current ages of the 94 PAHD patients ranged from 3 months to 15 years old. Among them, there were 62 children aged < 6 years with normal Gesell infant development scales (DQ ≥ 75) and 32 patients aged ≥ 6 years old. Of the ≥ 6-year-old patients, five were middle school students and 27 were primary school students. Based on the evaluation of their parents and teachers, academic performances at the middle to upper levels were seen in 28 children (5 MHP, 12 mPKU, 11 cPKU); two (both cPKU) showed academic performances at the lower level, mainly poor mathematics performance; and two (one cPKU and one mPKU) dropped out of primary school (IQ < 75 points) and had follow-up mean Phe concentrations of > 520 µmol/L.

## Discussion

Testing for HPA is included in neonatal screening in most parts of the world. Because early and regular treatment for HPA is correlated with better prognosis [9, 17], patients should be diagnosed and treated as soon as possible. In our study, we found that the primary treatment time was within 1 month after birth. BH4D accounted for only 2.21 % (4/181) of HPA cases, and all of them were PTPS deficient. Two patients died after cessation of treatment, and the remaining patients were presumed to have mild cases of the disease.

The incidence rate of PAHD varies worldwide, with incidences of 1/2,600 in the Turkish population [2], 1/4,500 in the Irish population [2], 1/10,000 in the European population [18], 1/125,000 in the Japanese population [2], and 1/15,923 in the Chinese population [19]. The incidence of HPA also varies widely throughout China, ranging from very low (1/188,679) in the southern regions to high (1/3,492) in the northern regions [19]. Nanjing in the central region of China has an incidence of about 1/6873 (Table 1). These results have updated the recorded PAHD incidence data for China and worldwide.

In China, p.Arg241Cys is frequently detected in Taiwanese patients, while p.Arg243Gln is common in both the northern and southern areas of the China mainland, which is consistent with our data [20, 21].

It has been previously reported that p.Arg53His may not be pathogenic [22]. Another report suggested that p.Arg53His may be a mild mutation, and there are several instances of cPKU or mPKU cases carrying this mutation [23, 24]. According to our hospital data, p.Arg53His was only found in MHP patients, but the serum Phe concentrations of patients carrying the p.Arg53His mutation all exceeded the cut-off value range and varied between 85 and180 µmol/L. We also performed extended carrier screening for 5,368 cases using hotspot mutation screening technology and discovered that 172 cases carried the c.158G > A mutation (two cases were homozygous) with a carrier rate of 3.2 %. Therefore, we believe that p.Arg53His is a pathogenic site and the mutation often causes MHP. No special dietary treatment is required with MHP, and the prognosis is good.

In our analysis of the follow-up data for the PAHD patients, we found that, although the cPKU and mPKU patients could control their Phe levels well by following the low-Phe diet, their levels were still higher than those of the MHP patients; therefore, it was difficult to reduce serum Phe further with dietary intervention. Whereas, even without dietary therapy, patients with MHP could maintain relatively low Phe levels compared with cPKU or mPKU patients.

In addition to low-Phe diet therapy, non-dietary treatments, such as sapropterin cofactor therapy and pegvaliase enzyme therapy, are also good choices for PAHD patients, as they have significant therapeutic effects [25–27]. Sapropterin cofactor therapy is mainly used for BH4D patients; however, 25–50 % of PAHD patients are also responsive to high-dose sapropterin cofactor; therefore, sapropterin is a treatment option for PAHD patients [27]. The major benefit of sapropterin cofactor therapy is that it increases dietary protein and Phe tolerance in responsive patients, allowing for the inclusion of more natural protein in the diet. Pegvaliase enzyme therapy is a new type of enzyme replacement therapy administered by subcutaneous injection that can reduce blood Phe independently of PAH and its BH4 cofactor [25]. However, in China, the use of sapropterin cofactor therapy or pegvaliase (Palynziq) enzyme therapy in PAHD patients is relatively limited, with the main consideration being the expense. Considering the high price and need for long-term use, and that non-diet therapy drugs have not been included in the scope of social medical insurance, it adds substantial economic pressure on the patient’s family, and many ordinary families struggle to afford the treatment. Furthermore, limited clinical experience in the use of the approach is also a consideration. Because non-diet treatments have been under gradual development in recent years, their history of clinical use is still relatively short and many treatments are still in the experimental stage. Combining the above reasons, the low-Phe diet is currently the most suitable treatment for PAHD patients in China.

When the preschool children (aged < 6 years old) and school-age children (aged ≥ 6 years old) were compared, there were significant differences not only between cPKU and mPKU patients but also MHP patients. This finding is easy to comprehend, as there were reported that the phe level of HPA patients who adhere to low-phe diet treatment will gradually increase with age [28]. Besides, the diets of school-age children are richer and more varied than those of preschool children, and it is more difficult for parents to strictly control their children’s diet once they have started school. The accumulation of excessive Phe and its metabolites in the body affects the function of the nervous system, leading to abnormal intellectual development [29], although patients who receive early diagnosis and treatment have a good prognosis. A large number of studies have shown that the level of intellectual development does not correlate with serum Phe concentrations at the initial screening and is mainly related to the subsequent control and stability of Phe concentrations [30]. In our research, most patients who insisted on follow-up treatment had normal intellectual development: they were able to study and undertake academic work. Only 13 patients, 11 of whom ceased treatment and two had poor treatment compliance, had intellectual deficiency and other complications. In addition, there were several patients whose ability to control their serum Phe concentrations was not ideal, but their intellectual development remained normal [31, 32]. Depending on the amount of Phe and metabolites that cross the blood–brain barrier, the brain Phe concentration may better reflect the relationship between Phe metabolism and intellectual development than the blood concentration [33, 34]. Exposure of the brain to high Phe concentrations can have detrimental effects on brain development and function, while similar serum Phe concentrations do not seem to have the same consequences in the brain [34, 35]. The mechanisms underlying these differences in intellectual outcomes among patients remain to be elucidated. In our study, two cPKU patients (a male aged 16 years and a female aged 18 years) had serum Phe concentrations that were not ideal, fluctuating around 800–1000 µmol/L, but they had healthy mental and physical development with excellent educational performance. However, this cannot be separated from the effects of additional home-based tutoring.

We also identified several cases of cPKU from other provinces in China without neonatal screening, and some cases showed manic and some depressive symptoms. Regardless of when the treatment is started, the symptoms can improve; for instance, one girl over 8 years old who had poor language ability went to school after a period of treatment. However, mental impairment is inevitable. Another patient over 16 years old was aggressive and self-harmed before treatment; however, after a period of treatment, the self-mutilation behavior decreased, indicating that even late treatment in patients is valuable [36, 37].

Maternal phenylketonuria (MPKU) was first proposed by Dent in 1957 [38]. Specifically, he reported three children born to PKU mothers who did not have PKU but were mentally retarded. Owing to the high level of Phe in the blood of female PKU patients before and during pregnancy, Phe and its metabolites (phenylpyruvate, phenyllactate, phenylacetic acid) enter the fetal blood circulation through the placental barrier to affect fetal development. The clinical manifestations include intrauterine growth retardation, microcephaly, congenital heart disease, intellectual deficiency, facial deformity, and even fetal death. An mPKU patient was detected by neonatal screening in 1989 and gave birth to a healthy baby in 2014 who was included in our study. The mother had received a strictly controlled diet of Phe concentrations at 120–360 µmol/L for 6 months before and during the pregnancy. Nutrition is one of the most important factors influencing pregnancy. However, strict dietary control can lead to a lack of nutritional elements and even a low birth weight and premature delivery. Therefore, developing an individualized nutritional management program is important, and the dietary management of the patients during pregnancy should be jointly supervised by genetic metabolism and nutrition specialists [39]. In some areas of China, neonatal screening is carried out earlier, when some female PKU patients reach childbearing age. However, given that many obstetricians and gynecologists do not know the significance of MPKU, it is necessary to further strengthen research in this area [40].

## Conclusions

HPA is preventable and curable. Most of the patients in our study achieved a good prognosis, which helped to improve their treatment compliance, and these results provide information to clinicians on the best treatment strategies. Our research into the development of PKU in Nanjing, Jiangsu, provides information on the latest clinical developments in southern Chinese cities and provides a reliable and updated database for future analysis and research into the genetic basis of PKU. In the future, we need to increase efforts in the screening, diagnosis, and treatment of HPA to reduce the severity of HPA and improve the quality of life of HPA patients.

## Methods

### Study population and data collection

From January 2001 to September 2020, we completed the neonatal screening of 1 292 622 newborns in Nanjing, Jiangsu Province of China. A total of 181 cases of HPA were diagnosed. The time point at which patient record collection began was defined as the baseline. The data retrospectively collected from electronic medical records included birthdate and sex, HPA diagnosis date and subtype, *PAH* genotype (if available), Phe concentration (reference range: 25–90 µmol/L), and Phe/Tyr ratio (reference range: 0.18–1.23) across the study period.

## Newborn screening

When the newborns were 48–72 h old with full lactation, about 200 µL of heel blood was collected to create a dried blood filter paper, and the concentrations of Phe and tyrosine (Tyr) were measured using a fluorescence quantitative method from January 2000 to November 2013 and tandem mass spectrometry from December 2013 onwards.

The study was reviewed and approved by the Ethics Committee of Nanjing Maternity and Child Health Care Hospital affiliated with Nanjing Medical University. Informed consent was signed by all parents.

## Diagnosis and differential diagnosis

HPA was diagnosed when Phe ≥ 120 µmol/L and/or Phe/Tyr > 2.0 on two occasions (initial screening and recall screening). All patients were diagnosed as soon as possible and underwent diagnosis examinations, including urinary pterin profile analysis, DHPR activity assay, and variant analysis. A coenzyme tetrahydrobiopterin (BH4) loading test was carried out in some cases.

## Variant analysis

Genomic DNA was extracted from peripheral venous blood taken from the patients and their parents using an OMEGA Genomic DNA Extraction Kit (OMEGA Bio-Tek, USA).

The full-length sequences of the 13 exons in *PAH* in patient genomes were analyzed from 2008 to 2013 using the ABI 3130 Gene Analyzer (Applied Biosystems, USA). We employed a second-generation sequencing panel from 2013 onwards, and library construction was performed using an Ion AmpliSeq Inherited Disease Panel Kit and an Ion AmpliSeq Library Kit (Life Technologies Inc., USA). Gene diagnosis panels were designed for inherited metabolic diseases. Panel 1 covered 18 diseases and 35 genes for amino acid metabolic diseases (including PAHD and BH4D). Panel 2 covered 17 diseases and 41 genes for organic acid metabolic and glycogen metabolic diseases. Panel 3 covered 15 diseases and 20 genes for fatty acid metabolic diseases. The three panels covered a total of 50 diseases and 96 genes. Sanger sequencing was performed on the PCR products to verify the results.

## Treatment and follow-up

A low-Phe diet containing sufficient calories and protein to meet the children’s growth and development requirements was given to the PAHD patients with serum Phe ≥ 360 µmol/L. BH4 (2–5 mg/kg), levodopa (5–15 mg/kg), and 5-hydroxytryptophan (5–10 mg/kg) were administered to BH4D patients. During the treatment, serum Phe concentrations were strictly monitored twice a week to once a month. Height, weight, head circumference, and other physical development indicators were also regularly monitored. The Gesell infant development scale (adaptability, fine action, big action, language, personal social interaction) was used for children aged < 6 years, and the Wechsler intelligent development scale (action, speech evaluation) was used for patients aged ≥ 6 years. DQ (developmental quotient) or IQ (intelligence quotient) was used to assess the intellectual development of the patients. When the DQ or IQ was lower than 75, the intellectual development was considered abnormal. Electroencephalogram (EEG) and cranial magnetic resonance imaging (MRI) were also used to measure brain activity every 1–2 years.

## Statistical analyses

Data are expressed as the median ± standard deviation. Independent Student’s t-test or two-way ANOVA test were used for statistic comparisons. Differences were considered significant when P < 0.05 (*, P < 0.05; **, P < 0.01; ***, P < 0.001).

## Supplementary Information

**Additional file 1.** Supplementary figures and tables.

## Data Availability

The datasets used and/or analyzed during the current study are available from the corresponding author on reasonable request.

## Abbreviations

HPA: Hyperphenylalaninemia
PAH: Phenylalanine hydroxylase
BH4: Tetrahydrobiopterin
PAHD: Phenylalanine hydroxylase deficiency
Phe: Phenylalanine
MHP: Mild hyperphenylalaninemia
mPKU: Mild phenylketonuria
cPKU: Classic phenylketonuria
PTPS: 6-Pyruvoyl-tetrahydropterin synthase
DHPR: Dihydropteridine reductase
HSP70: 70-kDa heat shock protein
PPV: Positive predictive value
MPKU: Maternal phenylketonuria
