# Supplementary material for: Neonatal screening and genotype-phenotype correlation of hyperphenylalaninemia in the Chinese population
Source: Orphanet J Rare Dis. 2021 May 12;16:214. doi: 10.1186/s13023-021-01846-w (PMC8114530; doi:10.1186/s13023-021-01846-w)
Supplement: Supplementary file 1 — Additional file 1. Supplementary figures and tables. [file 13023_2021_1846_MOESM1_ESM.pdf]

Supplementary Tables and Figures

Supplementary tables

Supplementary table 1. PPV of neonatal screening in recent years.

Year	Initial screening	Initial screening positives	Initial screening positive rate	Recalled	Confirmed	PPV
2015	83047	53	0.064%	53	9	16.98%
2016	90613	97	0.107%	97	5	5.15%
2017	98336	34	0.035%	34	4	11.76%
2018	87273	89	0.102%	89	13	14.61%
2019	83746	112	0.133%	112	4	3.57%
**2015-2019**	**443015**	**385**	**0.087%**	**385**	**35**	**9.09%**

PPV, positive predictive value

Supplementary table 2. Number and proportion of patients' clinical classifications

Classification		No. of cases	Initial screening Phe (μmol/L)	Recall review Phe (μmol/L)	Percentage
PAHD		177			97.79%
	cPKU	63	651.17 ± 308.92	1611.47 ± 532.61	34.81%
	mPKU	33	423.94 ± 130.96	725.44 ± 243.61	18.23%
	MHP	81	160.09 ± 51.74	170.84 ± 59.44	44.75%
BH4D		4	310.8±160.1	641.0±495.6	2.21%
Total		181			100%

Supplementary table 3. Urinary pterin profile analysis of HPA patients

Classification	Units	Urinary pterin profile	Reference ranges
neopterin	mmol/mol	10.4±8.1	1.2-2.92(newborn)
			0.90-7.49(2m-6m)
			0.29-2.61(6m-10y)
biopterin	mmol/mol	0.2±0.3	0.42-1.92(newborn)
			1.73-3.68(2m-6m)
			0.35-2.67(6m-10y)
biopterin/	%	2.3±0.4	19.8-50.3(newborn)
(biopterin+neopterin)			26.2-68.4(2m-6m)
			42.7-75.9(6m-10y)

Supplementary table 4. Information of the five died patients.

Index	Classification	Date of birth	Initial screening Phe (μmol/L)	Recall review Phe (μmol/L)	Time of death	Cause of death
1	cPKU	18/12/29	871.7	2057	<3	milk choking
2	cPKU	16/8/21	727	2330	<3	unknown (deny autopsy)
3	cPKU	16/8/15	493.76	2070	<3	unknown (deny autopsy)
4	cPKU	05/3/15	605	2964.5	-	unknown (family refuse to tell)
5	BH4D	14/2/8	393.25	1452	<3	unknown (deny autopsy)

<3*, Within 3 months after birth; -, unknown.

Supplementary table 5. Follow-up data over the last 4 years.

Classification	No. of patients	Mean Phe (μmol/L)	Mean Phe/Tyr ratio
cPKU	39	348.94 ± 25.15	7.03±4.64
mPKU	26	272.43 ± 21.31	4.89±2.94
MHP	29	169.97 ± 19.09	2.93±1.59
**PAHD**	**94**	**289.88 ± 15.64**	**5.30 ± 0.83**

Supplementary figures

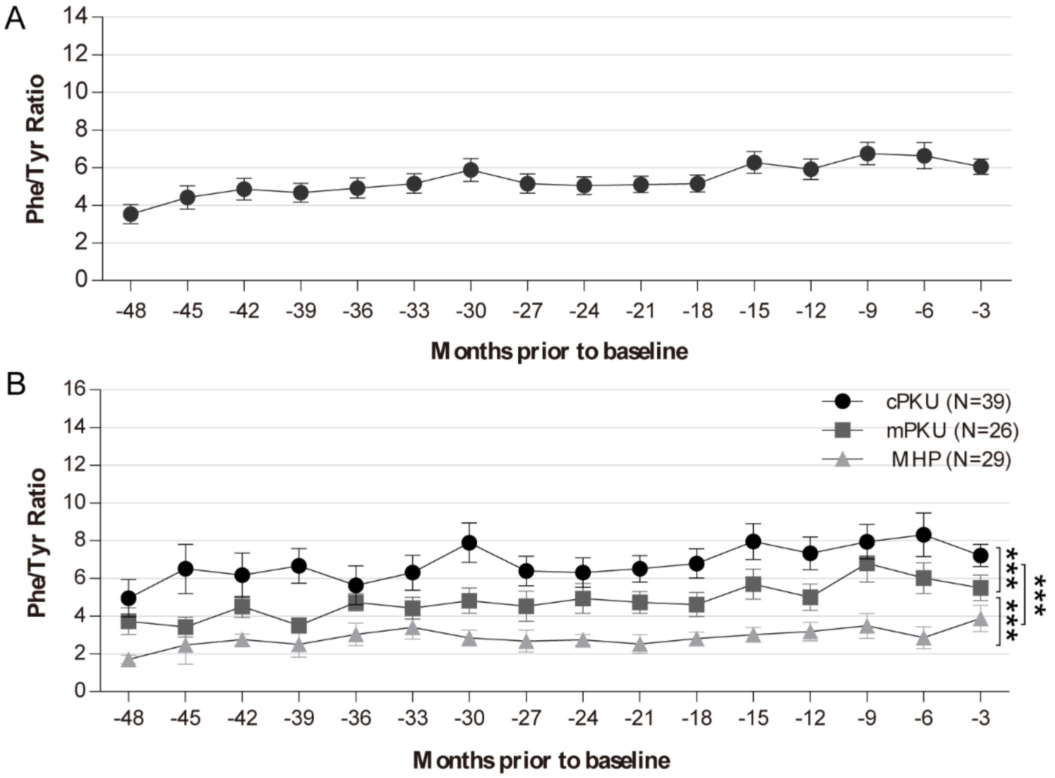

Supplementary figure 1. Mean serum Phe/Tyr ratio of PAHD patients over time across time windows of 3-month intervals. (A) Mean Phe/Tyr ratio of PAHD patients (n = 94) over time. (B) Respective mean Phe/Tyr ratios of cPKU, mPKU, and MHP patients over time. ***, P<0.001; error bars represent SEM.

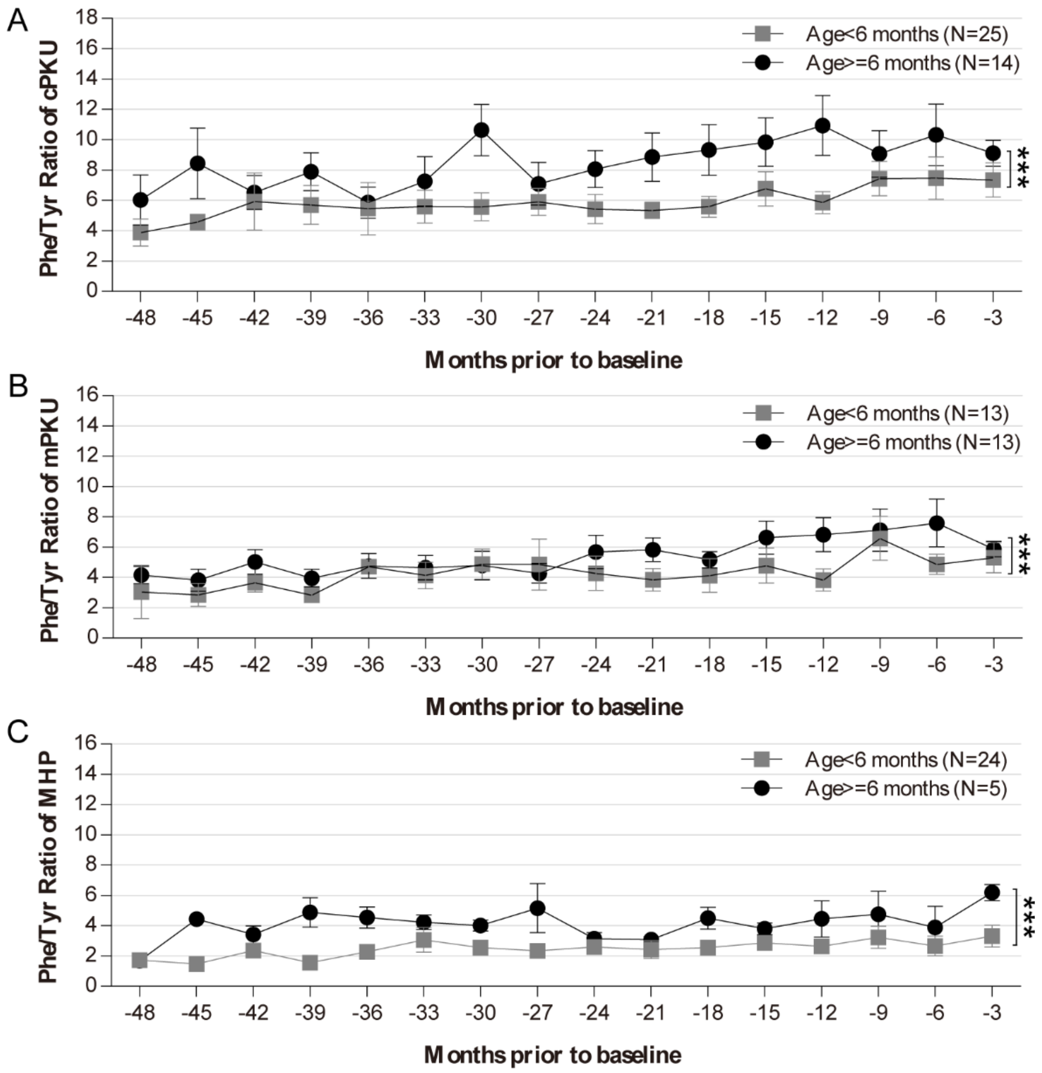

Supplementary figure 2. Mean serum Phe/Tyr ratios over time by age group (age <6 years vs. age ≥6 years) for cPKU (A), mPKU (B), and MHP (C) patients. ***, P<0.001; error bars represent SEM.
